# The Use of Acellular Dermal Matrix (Integra Single Layer) for the Correction of Malformative Chest Wall Deformities: First Case Series Reported

**DOI:** 10.1055/s-0042-1755622

**Published:** 2022-08-16

**Authors:** Carlos Delgado-Miguel, Miriam Miguel-Ferrero, Antonio Muñoz-Serrano, Mercedes Díaz, Juan Carlos López-Gutiérrez, Carlos De la Torre

**Affiliations:** 1 Department of Pediatric Surgery, La Paz Children's Hospital, Madrid, Spain; 2 Department of Pediatric Plastic Surgery, La Paz Children's Hospital, Madrid, Spain

**Keywords:** minimally invasive surgery, chest wall deformities, children, Poland syndrome

## Abstract

**Introduction** Autologous tissue transfers have been used in chest wall reconstruction for decades, with high morbidity. Recently, acellular dermal matrices (ADMs) have emerged as an alternative. The aim of this article is to report our initial experience in the reconstruction of malformative chest wall deformities with ADM.

**Methods** A prospective observational study was performed in patients with malformative chest wall deformities, who were reconstructed with ADM at our institution between 2018 and 2020. We analyzed demographic variables, surgical features, postoperative complications, and cosmetic results at 12 months' follow- up.

**Results** Four male patients were included (median age: 16 years). Two patients had bilateral costal anomalies, one patient had a unilateral chest deformity, and one patient had Poland syndrome. In all patients, blunt dissection of the subcutaneous cellular tissue overlying the defect was performed through 2.5 to 3 cm skin incisions, creating a pouch. Afterwards, several sheets of Integra Single Layer were placed in the pouch, to replace the volume defect. All patients were discharged same-day. No postoperative infections, hematomas, or seromas were observed. Only one patient presented with a partial surgical wound dehiscence. Revisions were performed at 1, 3, 6, and 12 months. All 4 patients were satisfied with the cosmetic outcome (Nuss Questionnaire: median score: 16 points; Q1–Q3: 22–26).

**Conclusion** The use of ADM in malformative chest wall deformities reconstruction has not been previously described in children. This study demonstrates that the use of ADM is a safe and reliable technique. However, more studies with long-term follow-up are warranted.

Autologous tissue transfers, such as muscle flaps and cartilage grafts, have been used in chest wall reconstruction for decades. However, these techniques result in an important donor-site morbidity, more significant in children due to their smaller bodies. Besides, sometimes donor sites might be scarce, of poor quality or lacking, such as in, for example, Poland syndrome, that might present with latissimus dorsi muscle and costal cartilage hipoplasia. 1 2 Recently, biological acellular collagen matrices, harvested from human or animal sources and processed for medical use, have emerged as an alternative to minimize donor-site morbidity. Ultimately, in most cases, the indication for correction of a chest wall deformity is cosmetic, and no function needs to be replaced, only soft tissue volume. When implanted, the acellular matrix triggers a remodeling process with cellular infiltration, neovascularization, and exchange of extracellular matrix 3 4 which, eventually, leads to the replacement of the defective tissue. 5

Acellular dermal matrices (ADMs) have been widely used for reconstructive surgery in many different anatomical areas, although data describing their use in chest wall reconstruction are limited. 6 In children, biological scaffolds have been predominantly used in abdominal wall reconstruction, while studies on their use for chest wall deformity correction are scarce. 7 8 To date, the use of ADM in chest wall reconstruction has only been reported in patients with primary chest wall malignancies, but not in congenital deformities, such as costal anomalies or Poland syndrome. 9 The aim of this article is to report our initial experience in the reconstruction of congenital chest wall deformities with ADM using a minimally invasive technique.

## Methods

A prospective observational study was performed in patients with congenital chest wall deformities, who were reconstructed with ADM at our institution between 2018 and 2020. Patients with chest wall deformities treated with other surgical techniques were excluded.

We analyzed demographic variables (gender, age at intervention, body mass index), personal history (previous diseases and surgeries, concomitant medical pathology, chronic treatment), surgical features (incision, number of ADM units implanted, operative time), length of hospital stay, postoperative complications, and cosmetic results, with pictures taken pre- and postoperatively. Cosmetic outcomes were also evaluated at 3 and 6 months postoperatively, using the Nuss Questionnaire, which is validated for chest wall deformities in children. 10 All patients and parents gave their consent to participate in the study as well as to take pictures of the affected areas for research. Photographs were taken protecting the identity of each patient. The study protocol was conformed to the guidelines of the 1975 Declaration of Helsinki and was approved by our institutional review board and by the hospital ethics committee.

For statistical analysis, data were collected in Microsoft Excel software version 2010 (Redmond, WA), and analyzed with SPSS Statistic version 22 (Chicago, IL). Categorical variables were expressed as frequency ( *n* ) and percentage (%). Continuous variables were expressed as means ± standard deviation if they were normally distributed, and those that were not normally distributed, as medians with interquartile range (Q1–Q3).

## Results

### Patients

Four male patients were included, with a median age of 15 years (Q1–Q3: 14–17), and a body mass index of 22 ± 2.4. Two patients had bilateral costal anomalies, affecting ribs 5 to 8. One case had a unilateral right chest deformity, affecting ribs 3 to 7. The fourth patient had a left Poland syndrome with complete major pectoralis muscle aplasia, congenital diaphragmatic hernia (which required a left thoracotomy and replacement with a goretex patch at birth), and an asymmetrical pectus excavatum (surgically corrected in 2016). Two patients (one patient with bilateral rib anomaly and the patient with Poland syndrome) had been previously treated with lipofilling in 2018, without managing to achieve a complete correction of the deformity due to the paucity of available donor site in patients with very scarce body fat and partial fat graft resorption.

### Operative Technique

**Fig. 1:**
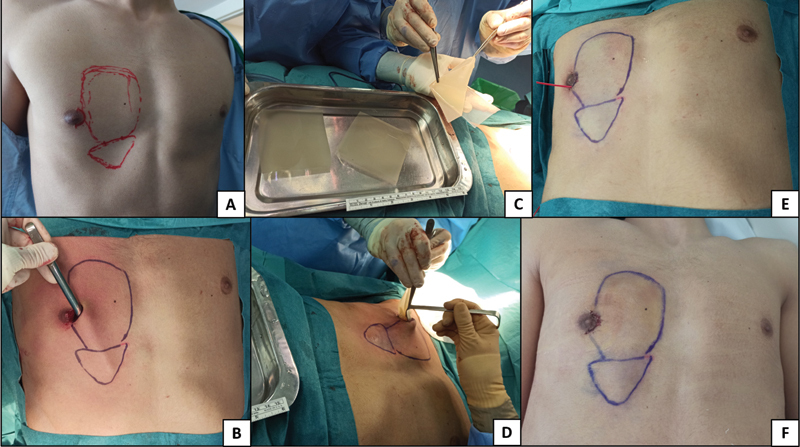
Operative procedure. ( **A** ) Preoperative marking of the defect. ( **B** ) Blunt dissection of the subcutaneous pouch through a periareolar incision. ( **C** ) Acellular dermal matrix (ADM) sheets (Integra Dermal Regeneration Template Single Layer) tailored to fit the defect. ( **D** ) Insertion of the ADM into the subcutaneous pouch. ( **E** ) Vessel-loop drain exteriorized through the incision. ( **F** ) Second postoperative day.

In all patients, 2.5 to 3 cm skin incisions were made 5 to 6 cm away from the chest wall defect. In one patient with bilateral rib deformity, one small oblique skin incision was performed bilaterally over rib 10. In the other patient with bilateral rib deformity, the defect could be corrected through a single mid-line longitudinal incision. The case with unilateral right chest deformity was approached through a lateral semicircular periareolar skin incision on the right nipple-areolar complex. In the patient with Poland syndrome, the skin incision was made over a previous central sternum scar used for the correction of the pectus carinatum. Subsequently, blunt dissection of the subcutaneous cellular tissue overlying the rib or muscle defects was performed, creating a subcutaneous pouch. At this point, and before introducing the ADM, hemostasia was achieved introducing into the subcutaneous pouch gauzes impregnated in an adrenaline solution (1 mg/L), which were left in place for 5 minutes. Afterwards, several sheets of ADM (Integra Dermal Regeneration Template Single Layer), previously cut into fragments tailored to fit and fill the defect of each patient were introduced through the incision and placed in the subcutaneous pouch in different directions, to fill and replace the volume defect in the chest wall. A vessel-loop drain, exteriorized through the incision, was placed in our first two cases (the patient with the Poland syndrome and the patient with the unilateral right chest deformity) and removed 48 hours afterwards in the outpatient clinic. Finally, the incision was closed in layers and a compressive dressing was applied. All patients were discharged the same day, at 6 to 8 hours postoperatively, with oral analgesia and instructions to remove the compressive dressing after 48 hours. The operative procedure is shown step by step in Fig. 1 .

### Outcomes

**Fig. 2:**
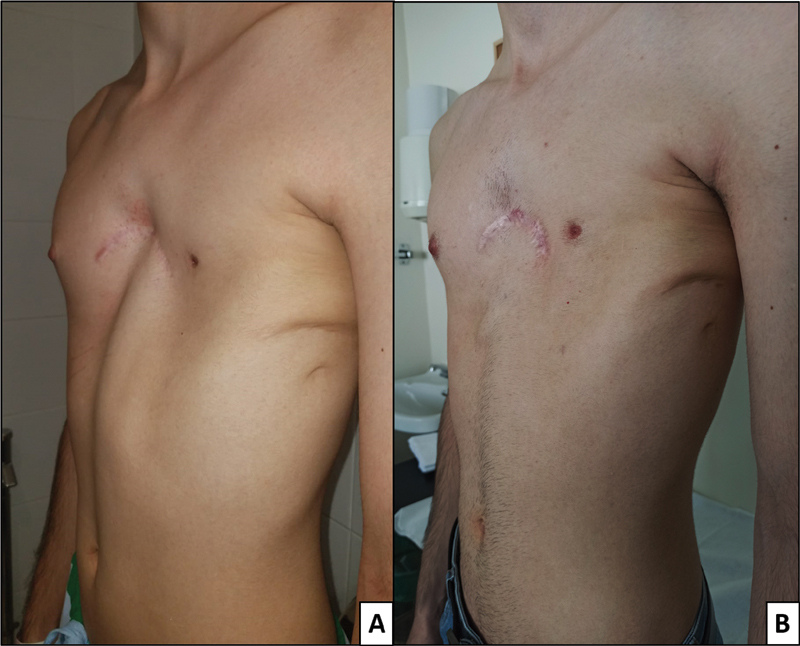
Poland syndrome with left pectoralis major muscle aplasia. ( **A** ) Preoperative. ( **B** ) At 12 months' follow-up.

**Fig. 3:**
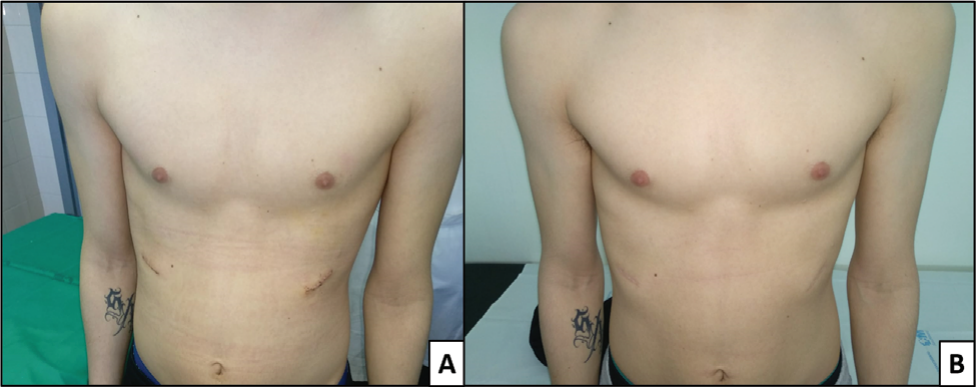
Patient with a bilateral costal malformation, affecting ribs 5–8. ( **A** ) At 7 days' follow-up. ( **B** ) At 12 months' follow-up.

The two patients with a drain were examined in the outpatient clinic on the second postoperative day; the other two patients, with no drain, were first examined 7 days after the intervention. At that time, surgical wound and initial cosmetic outcomes were both checked. No postoperative infections, hematomas, or seromas were observed. Only one partial dehiscence of the surgical wound was observed in one of the patients in whom a drain was placed, with no other postoperative complications, so in the ensuing patients no drain was left, with no complications arising. Subsequent revisions were performed at 1, 3, 6, and 12 months, when photographs were taken and the cosmetic result was evaluated ( Figs. 2 and 3 ). All 4 patients were satisfied with the cosmetic outcome at 12 months postoperatively. When analyzing the cosmetic outcomes using the Nuss Questionnaire, a median score of 16 points over a maximum score of 36 points (Q1–Q3: 12–18) was observed for the psychosocial items of the questionnaire, while for the physical items all patients had the highest score (12 points). Table 1 shows demographic, clinical, intraoperative features, and postoperative complications.

**Table 1:** Demographic, clinical, intraoperative features, and postoperative complications

Patient/Age	Congenital chest deformity	BMI	Previous surgeries	Approach	ADMs used (10 × 25 cm)	Drain	Postoperative complications
Male/17	Left Poland syndrome, left congenital diaphragmatic hernia (CDH), and pectus excavatum	19.6	CDH repair Pectus excavatum repair Lipofilling	Incision on previous central sternum scar	5	Yes	No
Male/16	Unilateral right chest deformity (ribs 3–7)	20.1	None	Lateral semicircular periareolar skin incision	3	Yes	Partial surgical wound dehiscence
Male/16	Bilateral rib anomaly 5 6 7 8	22.0	Lipofilling	Bilateral oblique incision over rib 10	4	No	No
Male/15	Bilateral rib anomaly 5 6 7 8	22.6	None	Single mid-line longitudinal incision	4	No	No

Abbreviations: ADM, acellular dermal matrix; BMI, body mass index.

## Discussion

This study is the first to report the use of ADM for the reconstruction of congenital chest wall deformities in children. The satisfactory cosmetic results in all patients, as well as the very low incidence of postoperative complications, make it a suitable technique in these patients. These promising initial results should be confirmed in series with larger numbers of patients and long-term follow-up.

Integra Dermal Regeneration Template Single Layer is an acellular porous matrix of cross-linked bovine tendon collagen and glycosaminoglycan. 11 The collagen-glycosaminoglycan biodegradable matrix provides a scaffold for cellular invasion and capillary growth and initially provides strength for tissue reinforcement while promoting rapid revascularization, white cell migration, and cell repopulation. This regenerative process ultimately leads to the replacement of the biological tissue matrix with native tissues.

The use of ADMs for chest wall reconstruction was first reported in 2004 by Cothren et al in adult patients. 12 Since then, the literature has remained sparse, with data limited to the use of other ADM different than Integra in case reports and a small series of patients. 13 To the best of our knowledge, this is the first published study in pediatric patients using ADM for congenital chest wall defects. The largest series reported to date consists of a retrospective review of 10 adults who underwent chest wall reconstruction with AlloDerm (five patients) or FlexHD (five patients). 14 Follow-up varied widely, ranging from 2 to 36 months, while in our study, the median follow-up is 12 months, because we started using this technique in 2018. However, the complication rate described by Ge et al in adult patients is higher than ours. Three of 10 patients developed wound seromas within 30 days, one with AlloDerm and two with FlexHD, with one seroma infection. In our series, only one partial surgical wound dehiscence was observed, with no other postoperative complications.

Our positive experience regarding texture, stability, durability, and resistance to infection with this type of biological implants for thoracic wall reconstruction are in accordance with the scarce published studies in children. 8 9 Lin et al reported the use of cross-linked porcine dermal collagen matrix in five patients who underwent resection for primary chest wall malignancy, with no postoperative mesh-related complications after a mean follow-up of 1.9 years, with results similar to those described in our study. 9 However, they did not report their cosmetic outcomes after surgery, so we cannot compare them with our results.

A satisfactory cosmetic result is probably the most important goal in the treatment of congenital chest wall defects such as Poland syndrome or costal anomalies, as these deformities do not generally cause physical symptoms, but generate an important psychological and emotional distress. 14 This explains why our patients obtained a maximum score on the physical items of the Nuss Questionnaire, for none of them had symptoms related to their deformity. In our opinion, the use of ADMs constitutes a safe and reliable alternative for the treatment of these deformities, with a satisfactory aesthetic result in all our patients.

In addition, it has several advantages over other materials and techniques previously used for chest wall reconstruction. In the past, the use of a wide variety of synthetic materials such as polypropylene, polytetrafluoroethylene, solid methyl methacrylate sandwich constructions, and various composite absorbable meshes has been explored. 15 The use of ADM has an advantage over prosthetic materials in the setting of contamination, because of its unique ability to undergo angiogenesis and integration into the host. 16 Since it is an acellular material, it does not incite inflammatory reactions and decreases the frequency and severity of adhesions. 17 As a biological graft, ADM integrate and incorporate within the host tissues, resulting in a soft, pliable, and strong newly formed tissue. The main advantage of ADM over other biological grafts or autologous tissue transfers, such as latissimus dorsi transfer, omental flap, or lipofilling, is the absence of donor-site morbidity. Furthermore, as mentioned in the introduction, donor sites might be scarce or lacking, such as in our patients, which presented with very low total body fat, which made lipofilling an unsuitable technique. Besides, in contrast to lipofilling, which usually requires several procedures to achieve the desired result due to partial fat graft resorption, ADM provide for a more stable and durable result.

Although these preliminary results are encouraging, it is mandatory to highlight a few limitations. First, our prospective study includes only 4 patients ant they are a very heterogeneous group. Second, the patients in our series have a median age of 15 years, so they had not yet achieved full growth at the time of operation, and their chest wall deformity might still progress in the following years. Finally, the follow-up period of 12 months is relatively short, so studies with larger numbers of patients and long-term follow-up are needed.

## Conclusion

The use of ADM in congenital chest wall deformities reconstruction has not been previously described in children. This study demonstrates that the use of a cross-linked bovine dermal acellular matrix is a safe and reliable technique for chest wall reconstruction in pediatric patients with malformative defects. Although it seems a promising technique, more studies with long-term follow-up are warranted.
